# Supplementary material for: Cell size reduction distinctly scales spindle elongation and chromosome segregation in C. elegans
Source: Nat Commun. 2026 Aug 6;17:7929. doi: 10.1038/s41467-026-76360-3 (PMC13448692; doi:10.1038/s41467-026-76360-3)
Supplement: Supplementary file 2 [file 41467_2026_76360_MOESM16_ESM.pdf]

nature portfolio

Peer Review File

Cell size reduction distinctly scales spindle elongation and chromosome segregation in *C. elegans*

Corresponding Author: Dr Chukwuebuka William Okafornta

This file contains all reviewer reports in order by version, followed by all author rebuttals in order by version.

Version 0:

Reviewer comments:

Reviewer #1

(Remarks to the Author)
Biological scaling has attracted biologists since Charles Darwin, and scaling of cell division remains one of the most compelling subjects in cell biology of scaling, as it can reveal key biophysical principles underlying embryonic control of cell division. In this manuscript, Okafornta et al. document spindle-length scaling and the lack of scaling in chromosome segregation in C. elegans embryos. By developing a machine-learning model, they quantitatively analyzed mitotic spindle dynamics up to the 64-cell stage. They found that spindle length scales with cell size, consistent with previous studies. In contrast, they report that chromosome segregation does not scale. Using precise laser ablation experiments, the authors conclude that chromosome segregation rate is not determined by kinetochore microtubules. The mechanism underlying the constant chromosome segregation rate remains unknown, but the authors speculate about a role for central spindle microtubule number based on electron tomography. Finally, they present a mathematical model for spindle-length scaling. Overall, this manuscript offers state-of-the-art imaging analysis and will be informative for C. elegans biologists. However, several conclusions are either redundant with previous research or inconsistent with findings in other systems. Furthermore, the mechanism underlying the constant chromosome segregation rate, a new biological phenomenon reported in this study, was not sufficiently investigated. Therefore, in its current form, the broader significance of this study for the general cell and developmental biology community remains unclear. The detailed comments follow below.

Major comments:
#1: The authors claim in the title that chromosome segregation rate does not scale with cell size, yet the data in Fig. 2g–j show moderate scaling. Therefore, the main claim of the paper does not appear to be fully supported.

#2: On conservation of chromosome separation rate scaling: Although the authors claim that chromosome separation rate does not scale with cell size, multiple studies in other organisms have reported scaling effect. In zebrafish embryos, chromosome separation rate scales with cell size (Afonso et al., Nature Cell Biology 2025; PMID: 39890956). In that study, anaphase A separation rate was constant, whereas anaphase B separation rate scaled with cell size (Fig. 1j–m), and cytoplasmic flow was identified as the mechanism underlying this scaling. In Drosophila embryos, both spindle size and chromosome separation speed scale with cell size (Xu et al., Current Biology 2025; PMID: 39793565). In yeast, chromosome segregation rate also scales with cell size (Krüger et al., eLife 2019; PMID: 30806623). Thus, chromosome segregation rate appears to scale with cell size across multiple organisms. In this context, the significance of the authors' finding is unclear without a clearly defined molecular mechanism. Moreover, as noted in comment #1, it is not evident that there is truly no scaling effect.

#3: In the first part of the manuscript, the authors describe cell-size–dependent scaling of spindle size and spindle elongation rate. However, these phenomena have already been reported and, to my knowledge, are not new (Hara and Kimura, Current Biology 2009; PMID: 19682904). The authors should explicitly clarify how their findings differ from, or add to, the previously published work.

#4: The mechanistic basis for why chromosome separation rate is independent of cell size remains unclear. The authors propose that a constant number of central spindle microtubules underlies this phenomenon, based on serial-section electron tomography. However, the tomography appears to have been performed on only one embryo each at the 8-cell, 16-cell, and 32-cell stages (a total of three samples). It is therefore uncertain whether these measurements represent a reliable statistical mean. Furthermore, no functional analysis is provided to investigate the mechanism that maintains a constant number of

central spindle microtubules. It would have strengthened the study if the authors had analyzed central spindle regulators, such as centralspindlin. On a related note, it has been reported that chromosome size scales with cell size (Ladouceur et al., JCB 2015: PMID 26033258; Zhou et al., eLife 2023: PMID 37096661). Given this, why the number of central spindle microtubules to remain constant despite the reduction in chromosome surface area?

#5: Line 223: “Our ablation experiments and electron tomography showed that such interpolar microtubules have little or no effect on the segregation dynamics of the chromosomes.”
A statistical test would be necessary to support this conclusion.

#6: On the mathematical model: The model appears to be an extension of previous studies (Hara and Kimura, Current Biology 2009; PMID: 19682904; Lacroix et al., Developmental Cell 2018; PMID29787710). Given that the spindle-length scaling result is not new, a mechanistic model addressing the mystery of the constant chromosome segregation rate would be more beneficial and better aligned with the novel aspects of the authors’ findings. Although the authors claim that “these simulation results show that regulation of microtubule dynamics over cell division can serve as a central mechanism for controlling both spindle scaling and chromosome separation through changes in cell size,” the data supporting the latter claim are not presented. In the model, the authors set the chromosome separation rate using the exponential function $\Delta c(t) = cf(1 - e^{(-t/\tau)})\,\hat{s}$, based on fitting to in vivo data. This equation does not appear to incorporate microtubule dynamics, which undermines the conclusion that microtubule dynamics regulate chromosome separation.

Minor comments:
#1: Line 191: “The motion of the chromatids, however, remained largely unchanged during
this time (Fig. 3e, coral).”
The immediate changes in Dp–p and Dp–c indicate that one centrosome becomes separated from the remaining part of the mitotic spindle. Because the central spindle remains intact, it is expected that Dc–c would not show an immediate change. The discrepancies between the rapid responses of Dp–p and Dp–c and the lack of an immediate change in Dc–c do not necessarily reflect differences in the forces driving their separation; rather, they indicate which components remain physically connected after the laser cut. In their previous work, the authors analyzed the long-term response to this laser cut and showed that it reduced the chromosome segregation rate (Yu et al., MBoC 2019, Fig. 5B), suggesting that interpolar microtubules contribute to chromosome segregation over a longer timescale.

#2: Line 200: “These results demonstrate that spindle pole dynamics at later stages are driven by pulling forces generated from cortically anchored motor proteins.”
The authors can infer the tension acting on kinetochore microtubules from this experiment. However, without severing the astral microtubules that connect the centrosome to the cortex, they cannot conclude whether this tension is generated by the cell cortex. Depletion of motor proteins or their regulators may be necessary to address this point.

#3: Line 205: “Previously, we combined such ablation experiments with electron tomography and showed that forces that segregate the chromosomes during the first mitosis in C. elegans are generated by a sub-population of microtubules nucleating and growing in between the chromosomes.”

If it is the same as the central spindle, it can be stated as such.
#4: C. elegans gene/sequence nomenclature:
According to C. elegans nomenclature guidelines (see section 9.2.5 of
https://www.wormbook.org/chapters/www_nomenclature/caenornomenclature.html), C27D9.1 appears to be the more appropriate designation than c27d9.1.

#5 Line 106: “The within-stage variance further confirms that our measurements can resolve lineage-specific variation.”
I think the strength of this new dataset lies in the lineage-resolved differences in spindle dynamics. The authors may consider presenting the actual lineage-specific differences as part of the data.

(Remarks on code availability)
I believe the authors reused previously developed code, and the original code was not reported in this paper. U-net model for image segmentation can be deposited as a resource for readers but is missing.

Reviewer #2

(Remarks to the Author)
During development, embryos typically undergo a series of cell divisions in which cell volume decreases by approximately half during each round of cell division. This manuscript uses divisions 2-7 of the C. elegans embryo as a model to explore how spindle dynamics vary as cells get smaller due to these cell size-reducing divsions. The authors combine light-sheet microscopy-based live filming with analysis of spindle structure by 3D EM-cryo-tomography, femtosecond laser ablation of microtubules between the spindle pole and chromosomes, and mathematical modeling to generate a picture of what happens to spindle dynamics during these divisions. Interestingly, they show that chromosomes segregate in a largely invariant fashion across these divisions(in which plots of the chromosome plate-to-plate distance over time can be overlaid) and suggest that this separation is driven by mid-spindle microtubules that form between the separating chromosomes. At the same time, they show that overall spindle length (pole-to-pole distance) is controlled by pulling forces on centrosomal microtubules exerted by cortical dynein motors and decreases in proportion to the reduction in cell size (which they distinguish from developmental stage). Using mathematical modeling, they show that they can recapitulate the change in

spindle length by varying only microtubule catastrophe frequency, with catastrophe frequency increasing as cell size decreases. The topic and findings of the manuscript are interesting and suitable for publication in Nature Communications. However, the manuscript would benefit from some textual revisions, as outlined below, to increase clarity and develop the story a bit more by including more explanation.
1. When describing the imaging used to capture spindle dynamics, including some additional details in the main text would be helpful. The fact that 3.8 million images were collected is not enough for the readers to really understand what was done here. How many embryos were imaged, how many z-sections were collected at each time point, what was the imaging time interval? What was the magnification? Although some of these details are mentioned later or can be gleaned from the methods, it is an important part of the data collection, and it would be helpful if the methodology were laid out more directly in the main text (and in the main figure).

2. The absence of needed details also made it more challenging to understand the next section on the scaling rules for spindle and chromosome dynamics. For example, the text says "We quantified spindle length and spindle elongation rate by fitting a sigmoid function and estimated cell size by calculating the cube root of cell volume for all wild-type datasets up to the 64-cell stage (see Methods). We observed that spindle length decreased by ~2-fold from the 2- to the 64-cell stage (~55 % decrease in length)". However, a variety of details needed to understand this are not included at this point in the text. In particular, I was wondering when spindle length measured. It would be worth detailing this a bit more. Something like, "The final length of the spindle determined using the sigmoidal fit was correlated with cell size. We observed…"

3. I also had a hard time understanding when the relevant aspect of spindle elongation that the authors are focusing on in their model (i.e. the increase that is not the increase in the chromosome-to-chromosome distance) occurs relative to anaphase onset. It also seemed like this aspect of elongation might start a bit earlier at the 2-cell stage than for the other stages.

4. The main focus of the manuscript is describing the component of spindle elongation that is not the increase in the chromosome plate-to-chromosome plate distance. It is quite hard for the reader to relate the segregation rate, which seems to be ~13 microns/minute over a very short time interval, to the overall elongation rate, which is between 2.5 and 4 over a longer interval (not well defined in the text). To make the point that the authors are aiming for and allow the reader to understand how the overall elongation rate relates to the segregation rate, it could be useful to break the pole-to-pole distance more clearly into two components. It might work well to present the analysis of the chromosome plate-to-chromosome plate distance, which is largely invariant across the divisions first. Then present the analysis of overall spindle length, which does vary with cell size. This could also include an analysis of overall spindle length minus the chromosome plate-to-chromosome plate distance, which would allow the reader to easily see what component of elongation can be attributed to the elongation of kinetochore microtubules versus the mid-spindle microtubules.

5. Based on cryo-electron microscopy, the authors describe the mid-spindle microtubules, which decrease in number from about 200 at the 1-cell stage to about 30 at the 32-cell stage. The authors postulate that the mid-spindle microtubules dictate the rate of chromosome segregation, yet they do not offer any explanation for how this relatively small population of microtubules would segregate the chromosomes in an invariant fashion. Can the authors amplify on this? Based on the images in Figure 3, these microtubules do not seem to form antiparallel bundles. Do they appear to interact with each other or with the interpolar microtubules that are in this region? More explanation of this population and how they might mediate invariant segregation is needed.

6. The authors suggest that an increase in catastrophe frequency as cell size decreases underlies the decrease in spindle length. It might be useful if the authors could speculate on some possible reasons why microtubule catastrophe frequency would increase as cell size decreases in the discussion.

(Remarks on code availability)

Reviewer #3

(Remarks to the Author)
Okafornta et al. quantitatively classify differences in the scaling of spindle elongation and chromosome separation during early C. elegans embryogenesis. They further demonstrate that the microtubules responsible for these processes, as well as their organization, differ qualitatively, using high-resolution electron tomography and nanoscale laser ablation techniques. Although models explaining cell-size-dependent spindle elongation already exist, the parameters of spindle dynamics and microtubule organization measured during embryogenesis in this study are novel. These findings are qualitatively convincing and provide important fundamental insights into a comprehensive understanding of spindle and chromosome dynamics during cell division, particularly with respect to cell-size dependency after the 2-cell stage. Nevertheless, some aspects of the quantification and discussion remain limited in their current form. The following points should be carefully addressed before publication.

Major comments:
1. In l.101 and onward, the authors discuss the contributions of lineages and developmental stages to variation in spindle dynamics using a random-effects model. However, there is no description of how the σ values were calculated or which parameters (e.g, spindle length, chromosome distance) were used in these calculations. Please clarify how these data were handled in the analysis. Regarding this point, it would also be helpful to present spindle and chromosome parameters for

each lineage, similar to Table S3, in which data are organized by developmental stage.
2. Throughout the manuscript, the authors demonstrate averaged values of spindle and chromosome parameters at each developmental stage. However, embryos at a given developmental stage contain multiple blastomeres with different cell and spindle sizes (e.g., large ABa, ABp, EMS blastomeres, and smaller P2 blastomeres in the 4-cell stage embryos). Given this size variation among blastomeres, both the average values and the distributions of individual parameters may be biased differently at each developmental stage. Could you provide detailed information on how the average values were calculated? Furthermore, to reduce the effects of size variation within a developmental stage, it might be more appropriate to limit the analysis to specific lineages with comparable cell sizes (such as using only AB lineage cells) when calculating and plotting average values.
3. The authors use a sigmoid function to fit spindle elongation dynamics across all developmental stages. While the dynamics in later developmental stages visually fit a sigmoid function, those in earlier stages appear to follow a different shape, such as an exponential curve (e.g., the 2-cell stage in wild-type embryos in Fig. S1). Is the use of sigmoid fitting appropriate for calculating the spindle elongation rates at all developmental stages? In relation to this concern, the calculated elongation rates may be somewhat lower than what is visually suggested by the graphs (e.g., in Fig.2, ~3 µm/ 30 seconds of elongation in 4-cell stage data).
4. From Fig. 2, the authors claim that chromosome separation remained mostly unchanged and is independent of cell size. The data support the notion that changes in chromosome separation parameters are smaller than those in spindle elongation and cell size during embryogenesis. However, the p-values of the slope shown in Table S4 indicate that chromosome separation does correlate with cell size. Please tone down and reframe the claim and the associated discussion accordingly. Furthermore, in Fig. 2g–i, the y-axis region from 0 to ~12 µm should be magnified to allow a more objective evaluation.
5. How could mid-spindle microtubules contribute to chromosome separation? The weak cell-size dependency of chromosome separation is an important finding of this study, and the authors are strongly encouraged to comment on possible underlying mechanisms.
6. Based on their simulation study, the cell-size-dependent catastrophe rate can account for the cell-size-dependent spindle elongation measured by the authors. This claim appears reasonable. Another important finding of this study is that the number of microtubules constituting the spindle and its components (interpolar microtubule and mid-spindle microtubules) differ. The effects of changes in microtubule numbers should also be evaluated using the simulation. To reproduce the observed cell-size dependency of spindle elongation, how many microtubules are required at each developmental stage under conditions of a constant catastrophe rate? Are the numbers comparable to the microtubule counts obtained from electron tomography?
7. As described in the Introduction section, centrosome and chromosome sizes also scale with cell size. Such size changes are expected to alter parameters related to drag forces and viscous friction, which are incorporated into the authors' model as a fixed parameter. Please discuss the potential effects of scaling of the spindle-associated structures themselves.
8. Please remove the description regarding possible applications to nuclear positioning and spindle formation in the Abstract (l.39–43) and Discussion (l.316–319). Because reference #18 is still in preparation, readers do not have access to the relevant information.

Minor comments:
1. From Figs. 3 and S6, the authors claim that chromosome separation is driven by the mid-spindle microtubules located between chromosomes. This conclusion would be strengthened by experiments assessing chromosome segregation in embryos with weakened cortical pulling forces (e.g., RNAi for gpr-1/2, goa-1, etc.).
2. The laser ablation between sister chromosomes and measurement of pole-to-chromosome distances in the uncut half of the spindle (shown in Supplemental Figure S3) are very useful data for discussing the microtubules responsible for chromosome separation. This important point should be described explicitly in the main text.
3. In the Methods section describing RNAi treatment, please include the IPTG concentration and the duration of incubation on RNAi plates. This information is essential for experimental reproducibility.
4. A previous study (Loughlin et al, 2011, Cell 147:1397–1407) identified katanin activity as a key scaling factor for metaphase spindle size. This may be related to the cell-size-dependent catastrophe rate modeled in the present study and would be worth discussing.
5. In Figs. 2d, 2e, 2h, and 2i, please ensure that the individual data points (shown in gray) are correctly represented. In the current figure, there appears to be no variation in cell size in these individual data, which may not reflect the real distribution, including different lineages data and intrinsic variation.
6. In l.203, "chromosome motion" should be replaced with "chromosome separation", because the chromosomes move after the laser ablation experiment shown in Fig. 3.
7. It would be helpful to clarify why data from the 1-cell stage were not included.

(Remarks on code availability)

Version 1:

Reviewer comments:

Reviewer #1

(Remarks to the Author)
The revised manuscript mainly involved text edits and did not specifically address the questions regarding biological

mechanism raised by this reviewer. Overall, I stand by my original assessment. While the new datasets may be useful as a resource, and the efforts made to generate the data should be commended, there are few new mechanistic insights presented in this manuscript. Aside from the generation of a new dataset, the main new biological information is that the chromosome segregation rate is less sensitive to the cell size change. However, the mechanism underlying this observation is not investigated in this manuscript. A more specialized journal seems more appropriate.

Major Comment #1:
Based on the authors' response and the revised title, both spindle length and chromosome separation rate scale with cell size, albeit to different extents. This is an interesting observation. However, what is the underlying mechanism? In other organisms, chromosome separation rate scales with cell size, which differs from those presented in this paper. In that case, what is the value of this new information outside the C. elegans research community? Without this question being answered, it remains difficult for me to assess the manuscript positively, as in my original assessment.

Major Comment #2:
In the rebuttal, the authors state: "A) In Hara & Kimura (2009), the authors only studied the spindle length dynamics up to the 8-cell stage. Considering the 3D geometry of the embryo and the 3D orientation of the spindle in different cells, we developed a 3D imaging and segmentation pipeline. First, we used lattice light-sheet microscopy that allows for gentle 3D multicolor long-term imaging of embryos up to the 64-cell stage. This then allowed us to perform precise and simultaneous measurements of both spindle dynamics and chromosome separation and determine cell volume. So, we significantly improved upon experimentation and microscopy."

As stated in my original assessment, I acknowledge the technical advancement of the present manuscript. However, given that spindle length scaling was already observed using data up to the 8-cell stage in 2009, confirmation of this rule up to the 64-cell stage in 2026 does not constitute a new discovery. This is why my assessment strongly focuses on the mechanism of the other new phenomenon: the scaling of chromosome separation distance.

Major Comment #3:
In the rebuttal, the authors state: "Although we did find roughly the same number of mid-spindle microtubules in our tomograms, we never explicitly mentioned a constant microtubule number."

It is not clear to me what information this tomography data is intended to convey if the authors did not draw any conclusions based on it. If it is simply meant to show the existence of the central spindle, it seems to represent only an incremental advance compared with their MBoC paper.

(Remarks on code availability)

Reviewer #3

(Remarks to the Author)
In this revision, the authors have satisfactorily addressed all of my concerns. The changes introduced by the authors during revision are reasonable.

(Remarks on code availability)

Open Access This Peer Review File is licensed under a Creative Commons Attribution 4.0 International License, which permits use, sharing, adaptation, distribution and reproduction in any medium or format, as long as you give appropriate credit to the original author(s) and the source, provide a link to the Creative Commons license, and indicate if changes were made.
In cases where reviewers are anonymous, credit should be given to 'Anonymous Referee' and the source.
The images or other third party material in this Peer Review File are included in the article's Creative Commons license, unless indicated otherwise in a credit line to the material. If material is not included in the article's Creative Commons license and your intended use is not permitted by statutory regulation or exceeds the permitted use, you will need to obtain permission directly from the copyright holder.
To view a copy of this license, visit https://creativecommons.org/licenses/by/4.0/
