# Supplementary material for: Cell size reduction distinctly scales spindle elongation and chromosome segregation in C. elegans
Source: Nat Commun. 2026 Aug 6;17:7929. doi: 10.1038/s41467-026-76360-3 (PMC13448692; doi:10.1038/s41467-026-76360-3)
Supplement: Supplementary file 3 [file 41467_2026_76360_MOESM1_ESM.pdf]

Cell size reduction distinctly scales spindle elongation and chromosome segregation in *C. elegans*

Chukwuebuka William Okafornta[1*⊥], Reza Farhadifar[2*⊥], Gunar Fabig[1], Hai-Yin Wu[3], Maria Köckert[1], Martin Vogel[1], Daniel Baum[4], Robert Haase[5,6], Michael J. Shelley[2, 7], Daniel J. Needleman[2, 3, 8], Thomas Müller-Reichert[1, 9⊥]

[1]Experimental Center, Faculty of Medicine Carl Gustav Carus, Dresden University of Technology, Dresden, Germany

[2]Center for Computational Biology, Flatiron Institute, New York, New York 10010, USA

[3]Department of Molecular and Cellular Biology, Harvard University, Cambridge, MA 02138, USA

[4]Department of Visual and Data-Centric Computing, Zuse Institute, Berlin, Germany

[5]Data Science Center, Leipzig University, Leipzig, Germany

[6]Center for Scalable Data Analytics and Artificial Intelligence (ScaDS.AI) Dresden / Leipzig, Leipzig, Germany

[7]Courant Institute, New York University, New York, New York 10012, USA

[8]School of Engineering and Applied Sciences, Harvard University, Cambridge, MA 02138, USA

[9]Core Facility Cellular Imaging, Faculty of Medicine Carl Gustav Carus, Dresden University of Technology, Dresden, Germany

[*]These authors contributed equally to this work

[⊥]Corresponding authors: chukwuebuka.okafornta@tu-dresden.de, rfarhadifar@flatironinstitute.org, mueller-reichert@tu-dresden.de

Supplementary Figures

Figure S1

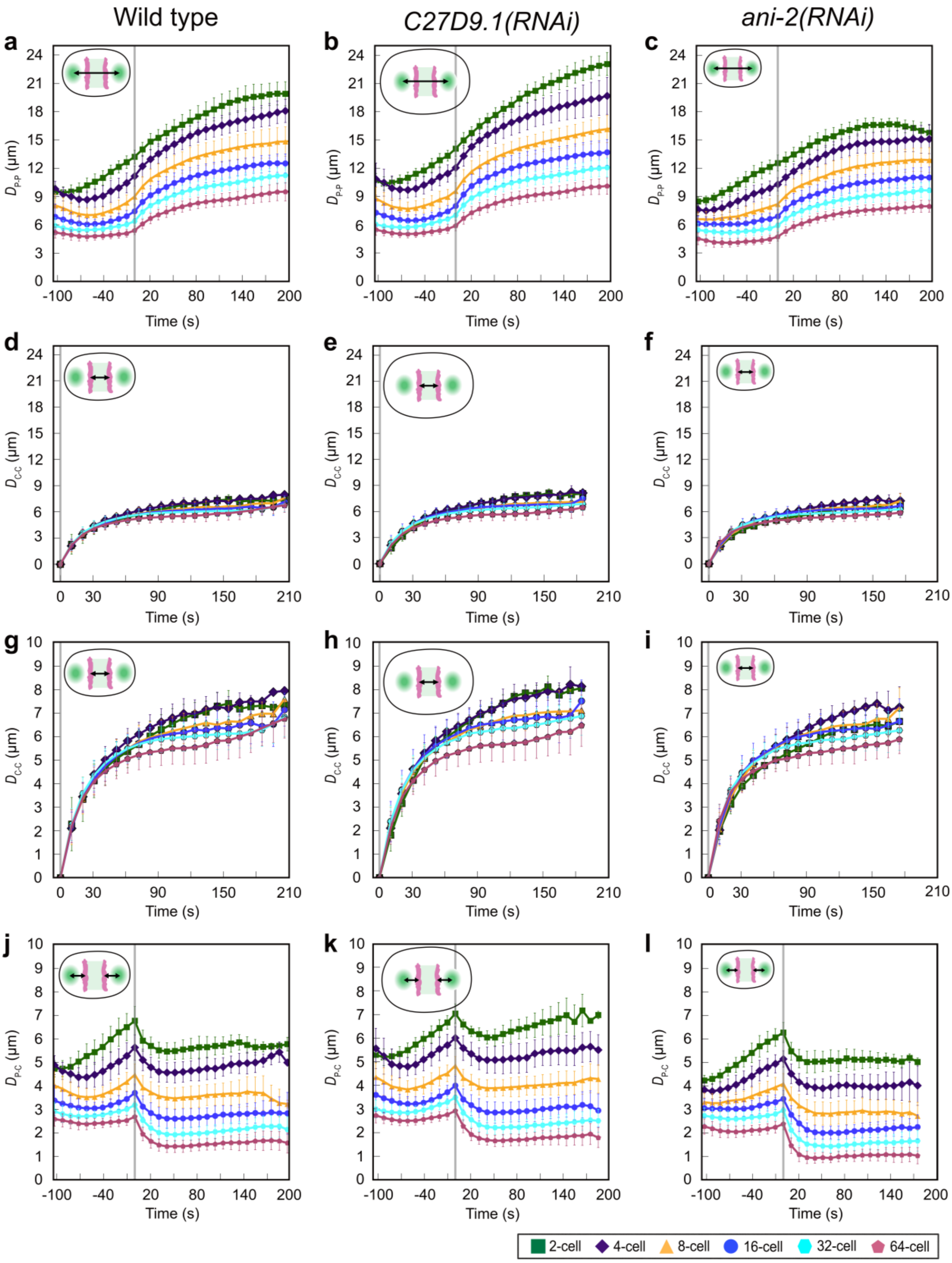

Figure S1| Spindle length and chromosome dynamics under different embryonic conditions. a-c, Pole-to-pole distance ($D_{P\text{-}P}$) given as a function of time for wild-type, *C27D9.1(RNAi)*, and *ani-2(RNAi)* embryos at different cell stages. Sample size (n) is indicated in Supplementary Table 1. **d-f,** Chromosome-to-chromosome distance ($D_{C\text{-}C}$) given as a function of time for wild-type, *C27D9.1(RNAi)*, and *ani-2(RNAi)* embryos at different embryonic cell stages. Sample size (n) is indicated in Supplementary Table 2. **g-i,** Zoomed-in plots of panels **d-f** showing chromosome-to-chromosome distance ($D_{C\text{-}C}$) as a function of time in wild-type, *C27D9.1(RNAi)*, and *ani-2(RNAi)* embryos. **j-l,** Pole-to-chromosome distance ($D_{P\text{-}C}$) as a function of time. Error bars represent the standard deviation of the corresponding measured distances. Each cell stage is indicated with a unique color code and symbol as shown in the plot legend. The grey vertical line in each panel at time = 0 indicates anaphase onset. The inset in the top left of each plot shows a schematic of the spindle, with the poles in green and chromosomes in magenta, and the black arrow indicates the measured distances.

Figure S2

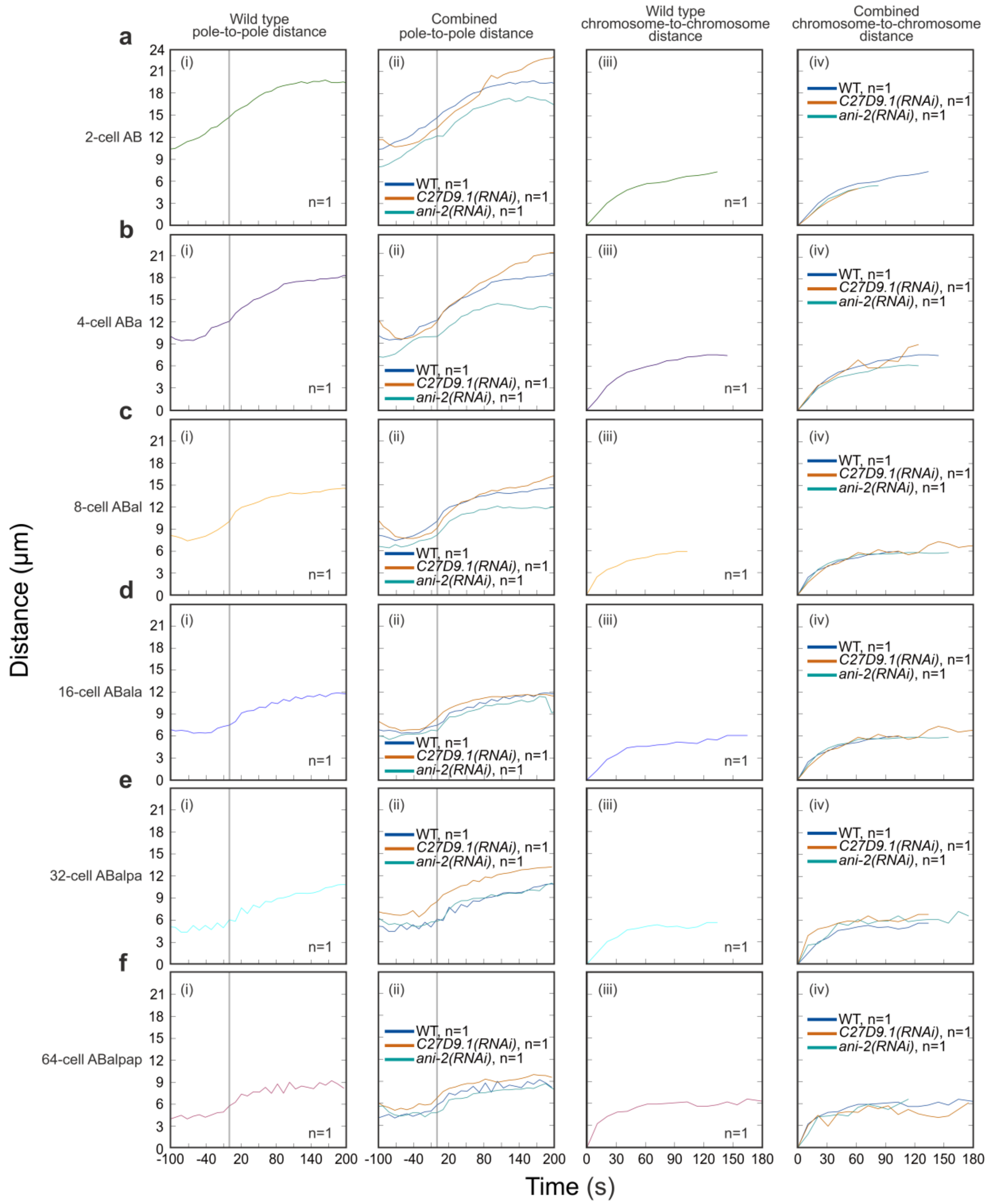

Figure S2| Cell-lineage resolved quantitative analysis of spindle dynamics in single embryos. a(i)**-f**(i)**,** Pole-to-pole distance given as a function of time in wild-type embryos. The 2-cell AB, 4-cell ABa, 8-cell ABal, 16-cell ABala, 32-cell ABalpa, and 64-cell ABalpap are shown (n = 1, for each cell type of each embryonic cell stage). **a**(ii)**-f**(ii)**,** Pole-to-pole distance given as a function of time for the cell types as shown in (a(i)-f(i)), for wild-type,

C27D9.1(RNAi) and *ani-2(RNAi)* embryos (n = 1, for each cell type of each embryonic cell stage). **a**(iii)**-f**(iii)**,** Chromosome-to-chromosome distance in wild-type embryos given as a function of time for a 2-cell AB, 4-cell ABa, 8-cell ABal, 16-cell ABala, 32-cell ABalpa, and 64-cell ABalpap (n = 1, for each cell type of each embryonic cell stage). **a**(iv)**-f**(iv)**,** Chromosome-to-chromosome distance given as a function of time for the cell types shown in (a(iii)-f(iii)) for wild type, *C27D9.1(RNAi)*, and *ani-2(RNAi)* embryos (n = 1, for each cell type of each embryonic cell stage).

Figure S3

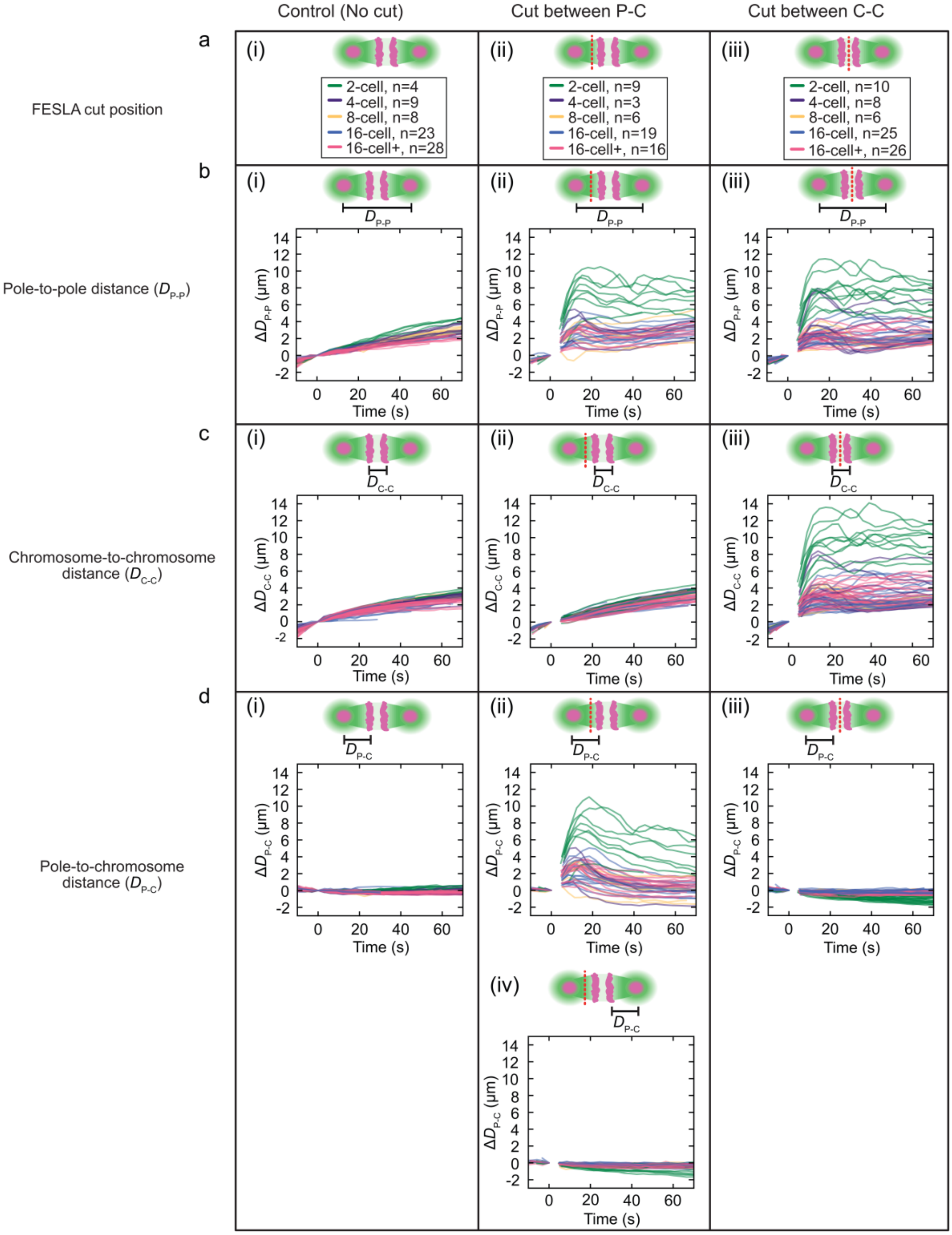

Figure S3| Laser ablation probes different forces in the early *C. elegans* embryo. a, Schematic drawing illustrating the positions of laser ablation on the mitotic spindle during FESLA experiments, where (i) shows the control when there is no laser cut on the spindle (n = number of sample for spindles in each cell stage with the corresponding color code), (ii) shows the laser cut (red dashed line) between the pole and the chromosomes in one half of the bipolar

spindle, (iii) shows the laser cut between the chromosomes. The microtubules are shown in green. **b,** Relative pole-to-pole distance ($D_{P\text{-}P}$) to the time of ablation cut at t = 0, given as a function of time. The black bar on the spindle schematics indicates the spindle region that is quantified on the corresponding plot. **c,** Relative chromosome-to-chromosome distance ($D_{C\text{-}C}$) to the time of ablation cut at t = 0, given as a function of time. **d,** Relative pole-to-chromosome distance ($D_{P\text{-}C}$) of the first half of the spindle given as a function of time. The relative pole-to-chromosome distance ($D_{P\text{-}C}$) in the second half of the spindle is shown in (iv), given as a function of time when there is a laser cut between the pole and chromosomes in the first half of the spindle.

Figure S4

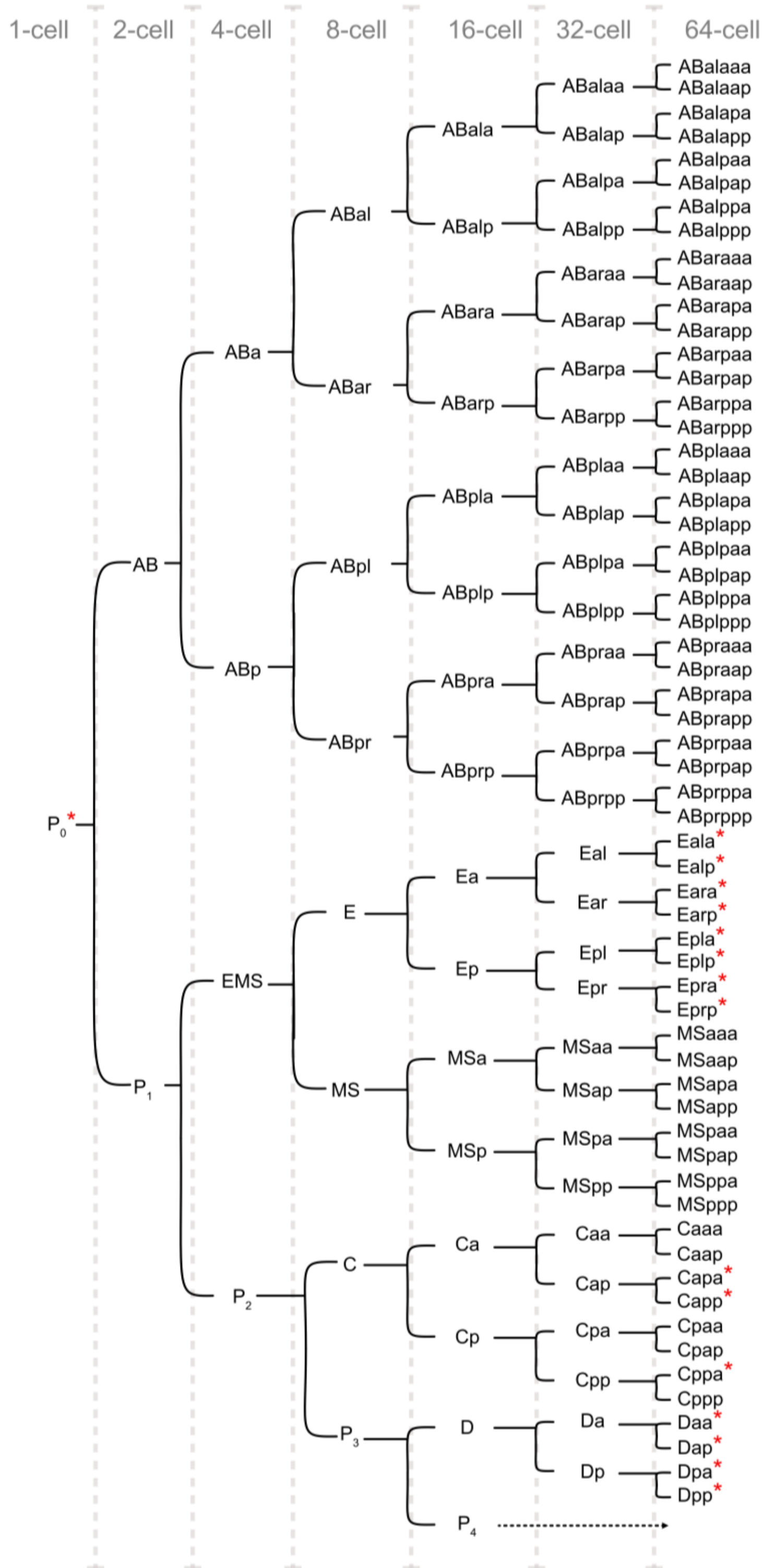

Figure S4| Mitotic lineage atlas for early *C. elegans* embryogenesis. The lineage atlas is based on Sulston et al. (1983)[1] and shows the identity of the cells in each embryonic cell stage whose spindles were analyzed. Cells marked with a red asterisk could not be analyzed, as cells within the center of the embryo were difficult to resolve. The 16-cell stage cell, P_4, does not go into division until the 120-cell stage (black dashed arrow).

Figure S5

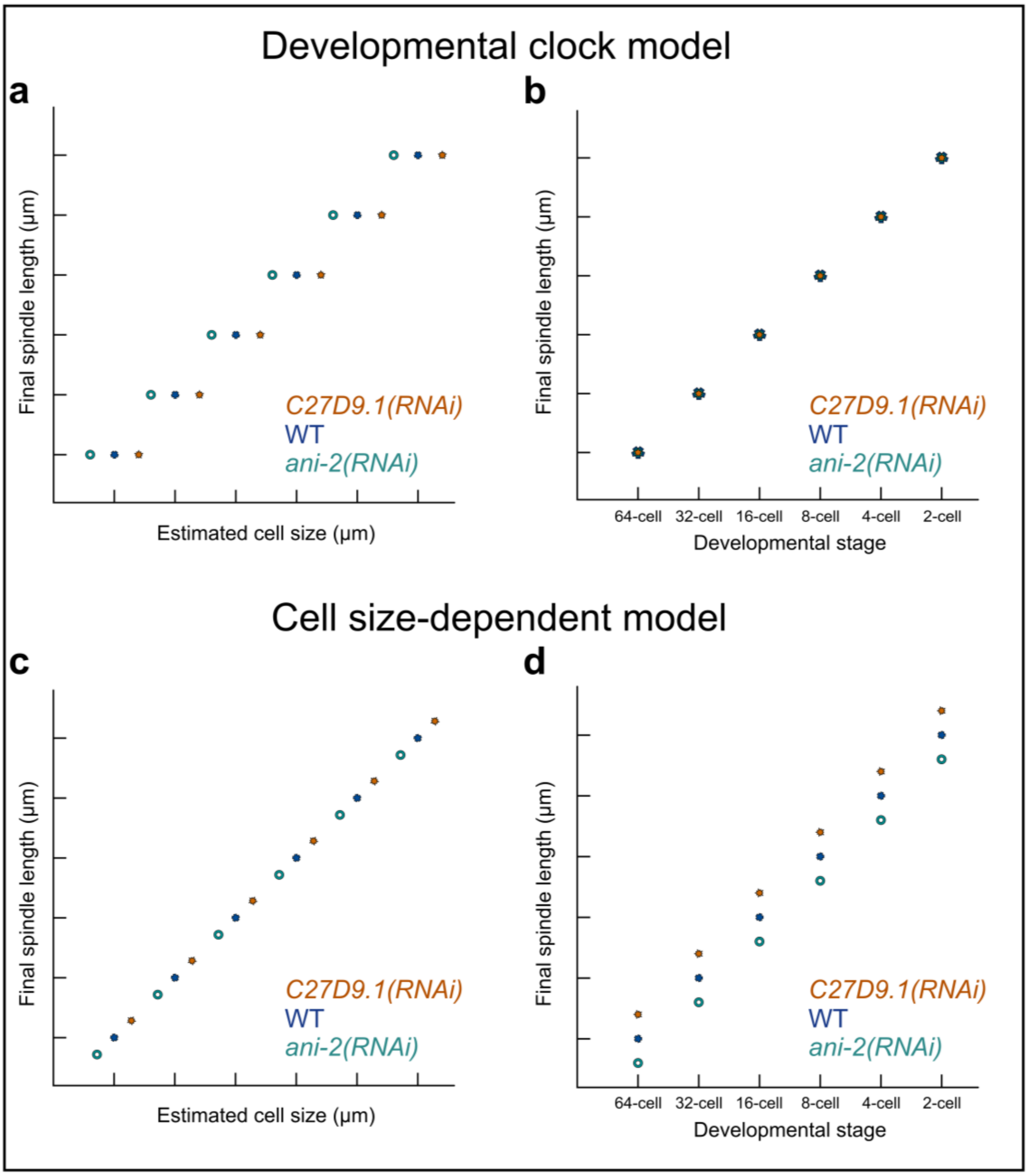

Figure S5| Models for spindle length dynamics. a,b, Developmental clock model showing (**a**) final spindle length plotted as a function of estimated cell size and (**b**) final spindle length plotted as a function of developmental stage for three embryonic conditions. **c,d**, Cell size–dependent model showing (**c**) final spindle length plotted as a function of estimated cell size and (**d**) final spindle length plotted as a function of developmental stage for the same three embryonic conditions. The three conditions are: wild type, blue; *C27D9.1(RNAi),* orange; *ani-2(RNAi)*, cyan.

Figure S6

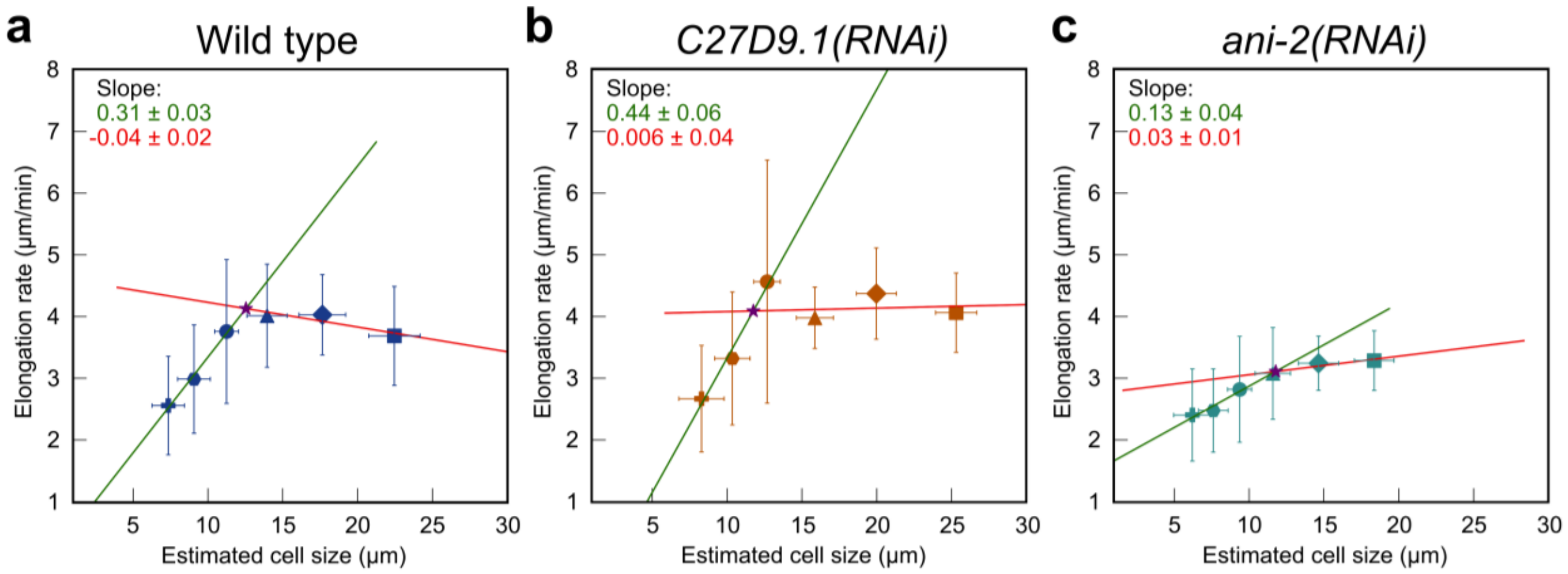

Figure S6| Spindle elongation rate as a function of cell size. Spindle elongation rates are plotted against cell size for **a,** wild type; **b,** *C27D9.1(RNAi)*; and **c,** *ani-2(RNAi)* embryos. Each plot shows a two-piecewise linear regression fit with corresponding slopes. The intersection point is indicated (purple star).

Figure S7

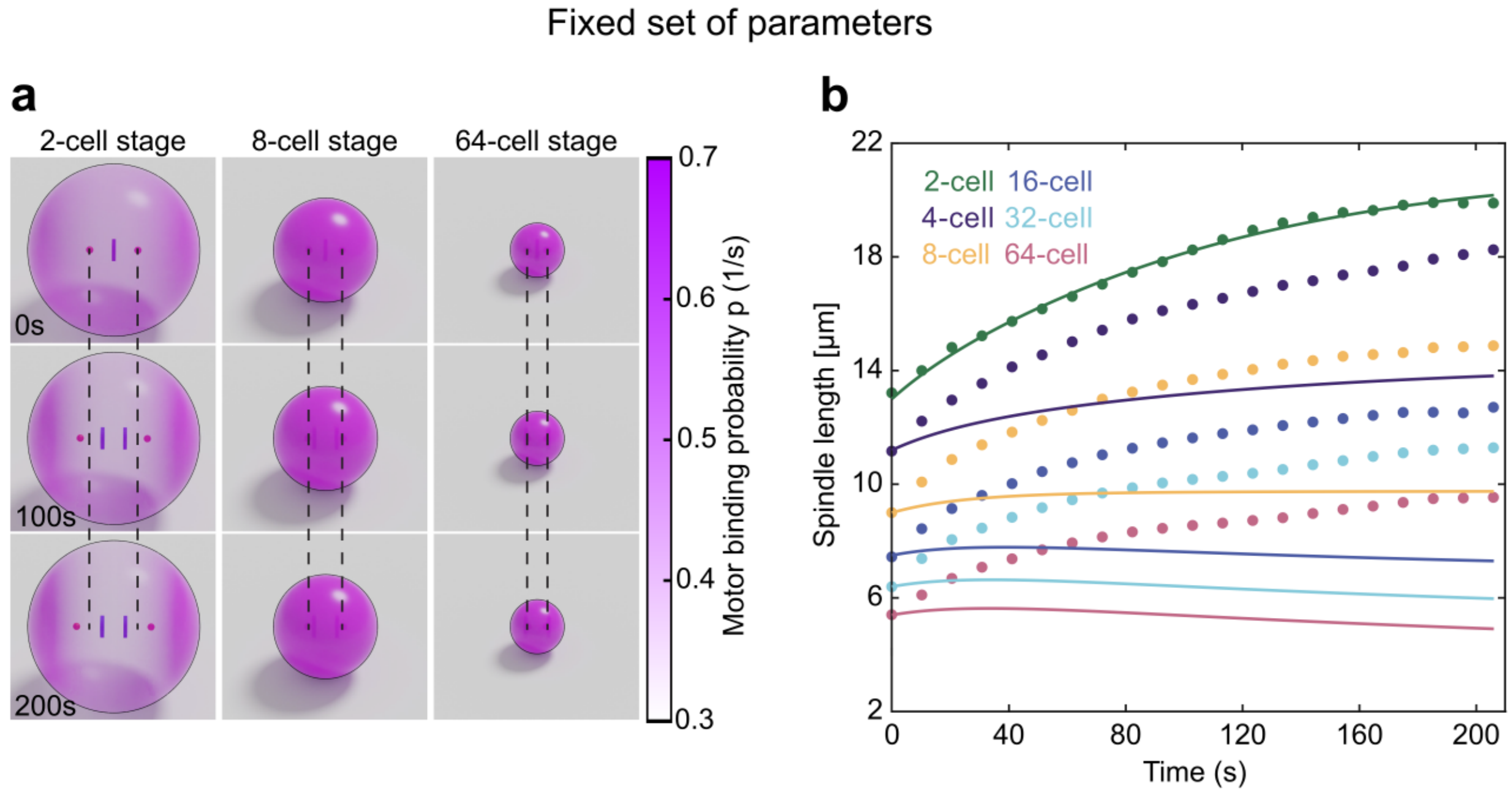

Figure S7| Model simulation for spindle length dynamics. a, Snapshots from simulations of 2-, 8-, and 64-cell stage cells. The binding probability of the motors is shown in pink. Dashed lines show the initial position of centrosomes. **b,** Spindle length as a function of time for different cell stages (lines show simulation, and dots indicate experimental measurements). The time t=0 corresponds to the onset of anaphase.

Figure S8

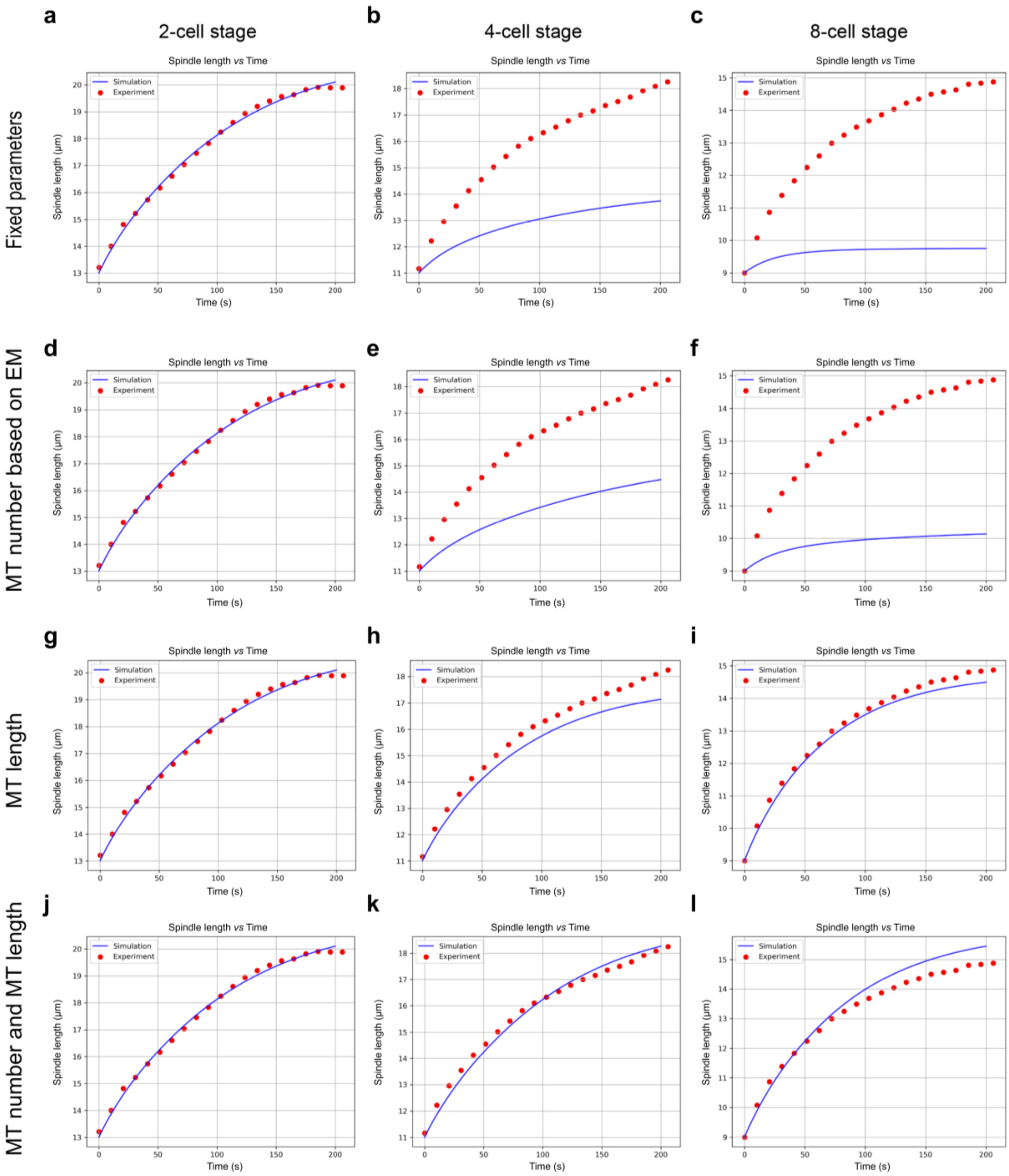

Figure S8| Extended parameter conditions for spindle length dynamics model simulation. Spindle length as a function of time for 2-, 4-, and 8-cell stage embryos showing the results from biophysical model simulations under different parameter regimes. **a-c,** All model parameters fixed. **d-f,** Microtubule number varied according to electron tomography measurements for each cell stage. **g-i,** Microtubule length varied**. j-l,** Both microtubule number and length varied. In all panels, the solid blue curves represent model simulations, and the red dashed curves represent experimentally measured spindle length dynamics from light microscopy. Time $t = 0$ corresponds to anaphase onset.

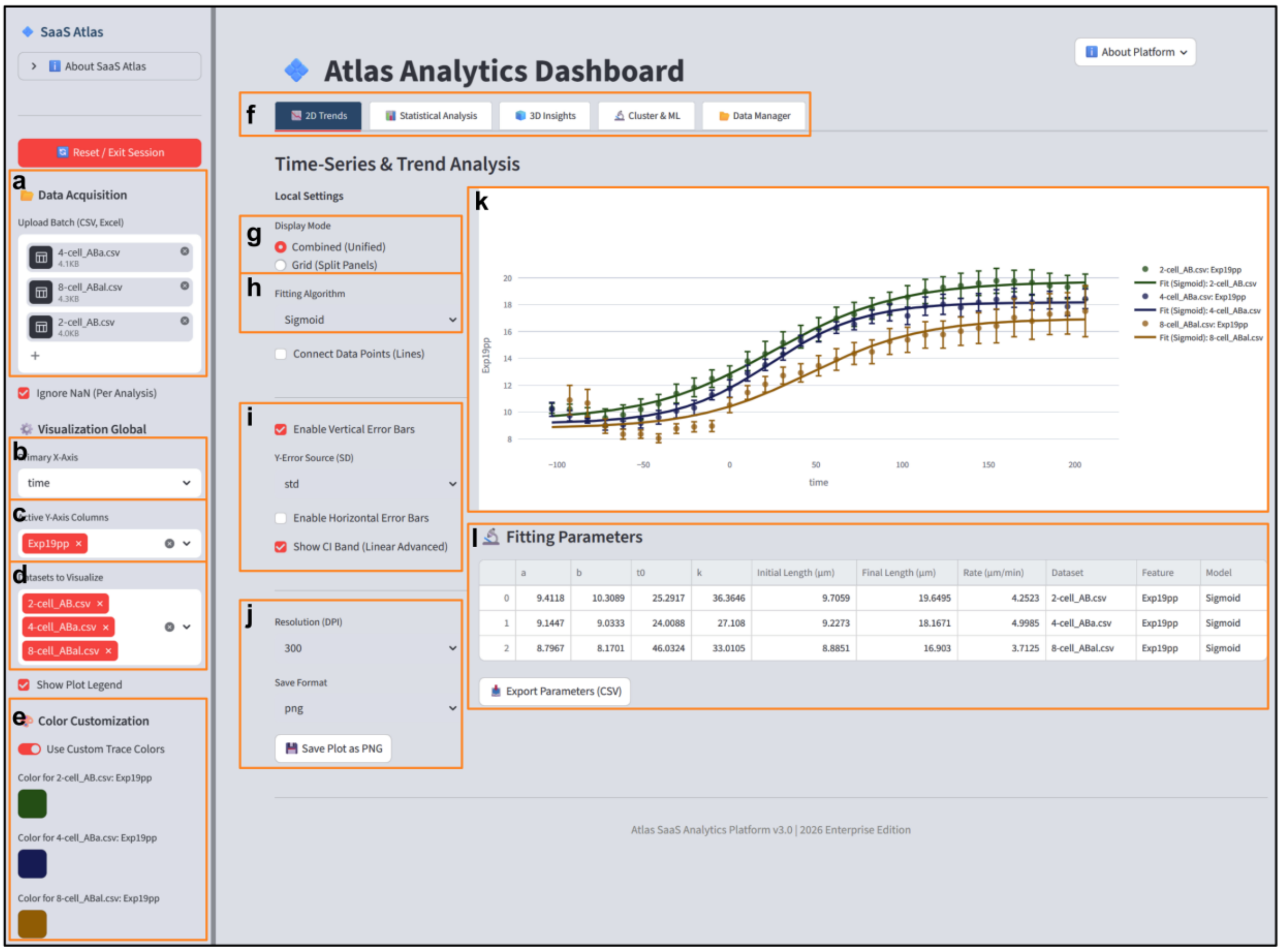

Figure S9| Graphic user interface for interactive data visualization. a, Data acquisition panel for importing batch datasets (CSV, Excel). **b,** Selection menu for primary x-axis parameters. **c,** Selection panel for active y-axis columns. **d,** Selection panel for datasets to be visualized. **e,** Color customization menu for individual data traces. **f,** Main navigation tabs including "2D Trends", "Statistical Analysis", "3D Insights", "Cluster & ML", and "Data Manager". **g,** Display mode selection for combined or split panel views. **h,** Fitting algorithm selection menu. **i,** Configuration panel for error bars and confidence interval (CI) bands. **j,** Export settings for image resolution and file format. **k,** Representative interactive visualization of time-series trends with fitted sigmoid models and error bars. **l,** Summary table of extracted fitting parameters with an option for CSV export.

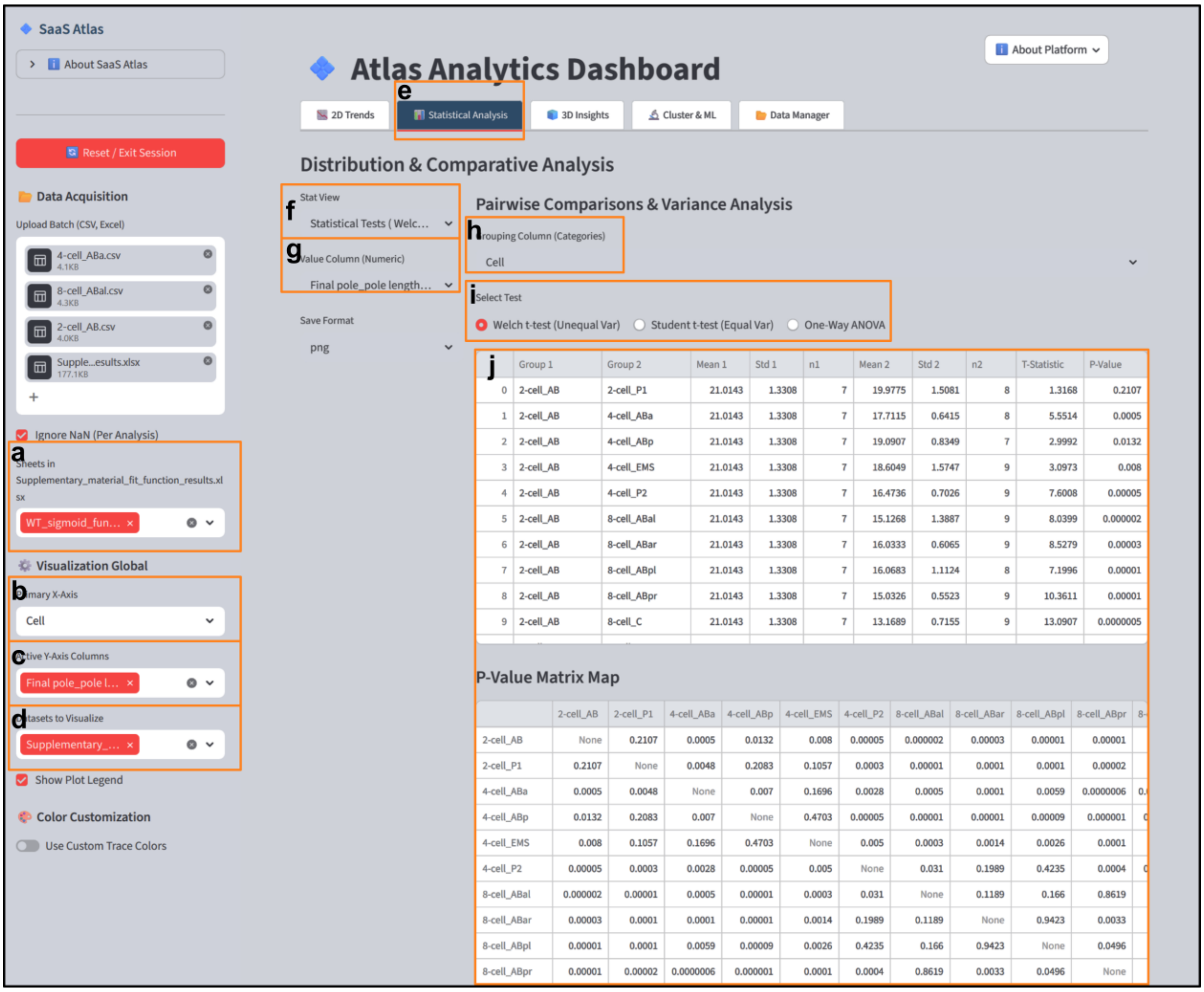

Figure S10| Statistical analysis module of the "Atlas Analytics Portal". a, Worksheet selection panel for multi-sheet Excel files. **b,** Selection menu for primary x-axis categorical grouping. **c,** Selection panel for numerical y-axis variables to be tested. **d,** Dataset selection panel for statistical processing. **e,** "Statistical Analysis" navigation tab. **f,** Statistical test selection menu. **g,** Value column selection for numerical data input. **h,** Grouping column selector for categorical variables. **i,** Test options including Welch's t-test, Student's t-test, and one-way ANOVA. **j,** Output display area featuring a summary results table and a corresponding p-value matrix map.

Supplementary Note (Graphic User Interface, GUI)

The GUI is a software-as-a-service dashboard called “Atlas Analytics Portal” that aids in interactive visualization of the mitotic spindle atlas dataset for spindle and chromosome dynamics listed in Supplementary Data 1. It is a browser-based software that can be accessed at https://saasdynamicatlas.streamlit.app/ and can also be run locally on Windows, Mac, or Linux systems by installing it from a Docker container in the GitHub repository (https://github.com/cwilliamOkafornta/SaaS_Dynamic_Atlas). The GUI is primarily designed for broad data visualization, interaction, mathematical fitting, and basic statistical test computation on the mitotic spindle atlas dataset. In addition, other tabular datasets from biological or biophysical experiments can be interactively visualized using this software, provided the input datasets are structured in rows and columns with column headers, as in the mitotic spindle atlas data structure. Supported file formats are CSV (.csv) and Excel (.xlsx).

To use the browser-based GUI, data files from any subfolder (e.g., WT_pole_to_pole) of the mitotic spindle atlas can be uploaded to the input panel by “drag and drop” (Fig. S9a). Each input file can be quickly inspected in the “Data Manager” tab (Fig. S9f). To visualize the data in an interactive plot, the x-axis parameter (e.g., time) should be selected from the drop-down bar in Fig. S9b. The x-axis can only plot one parameter. Also, the y-axis parameter should be selected, and multiple parameters can be plotted from the input files (Fig. S9c). The dataset to be visualized must be selected from the “Datasets to Visualize” panel (Fig. S9d). The plot legend can also be activated or deactivated as desired. Other features of the GUI include combined and split plots (Fig. S9g), color customization of the plots (Fig. S9e), implementation of error bars (Fig. S9i), and fitting mathematical functions such as sigmoid, exponential, linear regression, and support vector regression (Fig. S9h) to the plotted data. The data plots are shown in Fig. S9k. The plots can be saved as PNG, SVG, or PDF file (Fig. S9j). The parameters from the fitting are displayed on a table and can be saved as a CSV file (Fig. S9l).

A simple t-test can be performed using the dataset provided in the file *Supplementary_material_fit_function_results.xls*. The file should be uploaded via the “Data Acquisition” panel. The software automatically detects the available sheets, after which a sheet-selection panel becomes active (Fig S10a), where the desired sheet can be selected, for example, *WT_sigmoid_function_result*. To perform statistical analysis, the “Statistical Analysis” tab should be selected (Fig S10e). In the “Visualization Global” panel, the x-axis should be set to *Cell* (Fig. S10b), the y-axis to *Final pole_pole length (µm)* (Fig. S10c), and the dataset to be visualized should be selected as *Supplementary_material_fit_function_results.xlsx* (Fig. S10d). Statistical testing is then

enabled in the panel shown in Fig. S10f, the parameter *Final pole_pole length (µm)* is selected in Fig. S10g, and the desired test is chosen in Fig. S10i. The results are presented both as a table and a matrix map (Fig. S10j)

Supplementary Tables

Table S1| *P*-values for the pole-to-pole elongation rate derived from Welch's two-sided t-test for the analyzed embryonic conditions.

Wild type						
	2-cell	4-cell	8-cell	16-cell	32-cell	64-cell
2-cell	1					
4-cell	0.17312239	1				
8-cell	0.18201251	0.920051	1			
16-cell	0.76458859	0.080965	0.0751	1		
32-cell	0.00612913	1.33E-10	1.38E-14	3.41E-10	1	
64-cell	0.00010947	3.46E-15	1.48E-23	1.62E-21	4.69E-08	1
n	15	33	70	135	213	279
C27D9.1(RNAi)						
2-cell	1					
4-cell	0.243987	1				
8-cell	0.714287	0.014885	1			
16-cell	0.094467	0.422763	0.005316	1		
32-cell	0.007267	3.83E-08	1.51E-09	3.10E-08	1	
64-cell	7.57E-05	9.05E-14	1.22E-31	9.56E-16	2.76E-10	1
n	10	29	58	102	180	208
ani-2(RNAi)						
2-cell	1					
4-cell	0.001966	1				
8-cell	1.92E-08	2.56E-10	1			
16-cell	8.61E-09	1.56E-17	3.83E-19	1		
32-cell	3.50E-09	4.55E-21	4.18E-36	7.10E-33	1	
64-cell	1.68E-09	1.30E-23	1.88E-45	1.46E-72	4.80E-55	1
n	8	30	62	107	157	148

Table S2| *P*-values for the chromosome-to-chromosome segregation rate derived from Welch's two-sided t-test for the analyzed embryonic conditions.

Wild type						
	2-cell	4-cell	8-cell	16-cell	32-cell	64-cell
2-cell	1					
4-cell	0.80017772	1				
8-cell	0.93054264	0.634831	1			
16-cell	0.89190577	0.18432	0.452379	1		
32-cell	0.37002153	0.000208	0.00115	0.000597	1	
64-cell	0.5083043	0.001862	0.009554	0.009784	0.232402	1
n	17	30	65	130	206	263
C27D9.1(RNAi)						
2-cell	1					
4-cell	0.01236	1				
8-cell	0.000853	0.193446	1			
16-cell	4.16E-06	0.001115	0.033454	1		
32-cell	1.48E-06	3.53E-06	0.001017	0.672624	1	
64-cell	0.000103	0.010033	0.334798	0.092195	0.000827	1
n	11	26	52	98	174	204
ani-2(RNAi)						
2-cell	1					
4-cell	0.007965	1				
8-cell	0.002069	0.687348	1			
16-cell	1.27E-05	0.003707	0.002827	1		
32-cell	1.37E-07	4.79E-07	1.34E-08	0.002517	1	
64-cell	1.32E-09	1.72E-08	1.16E-09	8.47E-05	0.140927	1
n	8	31	62	113	148	126

Table S3| Summary of the *C. elegans* mitotic spindle dynamics parameters.

	2-cell	4-cell	8-cell	16-cell	32-cell	64-cell
**Wild type**						
**Cell volume (µm³)**	11337.00 ± 2597.08	5499.32 ± 1455.04	2715.33 ± 784.27	1423.68 ± 302.11	745.53 ± 268.61	397.08 ± 175.15
**Estimated cell size (µm)**	22.46	17.65	13.95	11.25	9.07	7.35
**Error propagation**	1.72	1.56	1.34	0.80	1.09	1.08
**Initial pole-to-pole distance (µm)**	7.38 ± 1.91	8.38 ± 0.87	7.01 ± 0.86	5.87 ± 1.13	5.20 ± 0.65	4.67 ± 0.44
**Final pole-to-pole distance (µm)**	20.46 ± 1.52	17.91 ± 1.43	14.57 ± 1.47	12.44 ± 0.89	11.01 ± 0.76	9.29 ± 0.88
**Final chromosome-to-chromosome distance (µm)**	6.89 ± 0.73	7.14 ± 0.53	6.54 ± 0.65	6.33 ± 0.61	6.10 ± 0.56	5.6 ± 0.67
**Metaphase pole-to-pole distance (µm)**	13.27 ± 0.77	11.27 ± 1.06	9.13 ± 0.86	7.66 ± 0.67	6.78 ± 0.43	5.71 ± 0.36
**Elongation rate of poles (µm/min)**	3.69 ± 0.80	4.03 ± 0.65	4.01 ± 0.84	3.76 ± 1.17	2.99 ± 0.88	2.56 ± 0.80
**Segregation speed of chromosomes (µm/min)**	13.69 ± 8.90	13.10 ± 3.05	13.49 ± 4.63	14.00 ± 4.06	15.76 ± 5.18	15.20 ± 4.78
ani-2(RNAi)						
**Cell volume (µm³)**	6195.37 ± 1342.83	3137.68 ± 867.48	1556.91 ± 475.71	822.81 ± 213.12	441.56 ± 171.12	239.37 ± 143.89
**Estimated cell size (µm)**	18.37	14.64	11.59	9.37	7.61	6.21
**Error propagation**	1.33	1.35	1.18	0.81	0.98	1.24
**Initial pole-to-pole distance (µm)**	7.52 ± 0.75	6.67 ± 1.80	6.23 ± 0.83	5.78 ± 0.79	5.01 ± 0.68	4.04 ± 0.43
**Final pole-to-pole distance (µm)**	16.60 ± 0.67	15.33 ± 1.41	12.93 ± 1.17	11.02 ± 0.91	9.44 ± 0.83	7.83 ± 0.58
**Final chromosome-to-chromosome distance (µm)**	6.36 ± 0.42	6.72 ± 0.69	6.54 ± 0.58	6.33 ± 0.70	5.93 ± 0.57	5.37 ± 0.52
**Metaphase pole-to-pole distance (µm)**	12.70 ± 0.89	10.55 ± 0.81	8.50 ± 0.88	7.20 ± 0.69	6.16 ± 0.50	4.91 ± 0.37
**Elongation rate of poles (µm/min)**	3.29 ± 0.48	3.24 ± 0.44	3.08 ± 0.74	2.82 ± 0.86	2.48 ± 0.68	2.40 ± 0.75
**Segregation speed of chromosomes (µm/min)**	11.03 ± 1.43	13.23 ± 2.86	13.50 ± 3.13	15.18 ± 4.08	16.88 ± 4.88	17.90 ± 6.24
C27D9.1(RNAi)						
**Cell volume (µm³)**	16228.51 ± 2647.68	7973.19 ± 1594.60	4000.03 ± 934.68	2037.04 ± 428.12	1111.67 ± 380.67	570.53 ± 311.46
**Estimated cell size (µm)**	25.32	19.98	15.87	12.68	10.36	8.29
**Error propagation**	1.38	1.33	1.24	0.89	1.18	1.51
**Initial pole-to-pole distance (µm)**	8.58 ± 1.07	9.29 ± 1.22	7.51 ± 0.84	6.23 ± 1.14	5.36 ± 0.78	4.87 ± 0.44
**Final pole-to-pole distance (µm)**	23.72 ± 1.20	19.56 ± 2.02	15.75 ± 1.35	13.45 ± 1.13	11.77 ± 0.83	9.90 ± 0.74
**Final chromosome-to-chromosome distance (µm)**	7.99 ± 0.53	7.62 ± 0.60	6.87 ± 0.49	6.81 ± 0.78	6.43 ± 0.56	5.78 ± 0.68
**Metaphase pole-to-pole distance (µm)**	14.16 ± 0.65	12.42 ± 0.98	9.90 ± 0.90	8.26 ± 0.73	7.36 ± 0.43	6.17 ± 0.43
**Elongation rate of poles (µm/min)**	4.06 ± 0.64	4.37 ± 0.74	3.98 ± 0.50	4.57 ± 1.97	3.32 ± 1.08	2.67 ± 0.86
**Segregation speed of chromosomes (µm/min)**	11.43 ± 1.81	13.39 ± 2.16	14.23 ± 3.36	15.86 ± 5.92	16.15 ± 4.18	14.75 ± 3.72

Table S4| Comparison of parameters of mitotic spindle dynamics in *C. elegans* for the three embryonic conditions. P-value of the slope for each spindle trait is derived as a two-sided t-test on the regression slope with degrees of freedom = n – 2 (n: number of cell length data points).

	Spindle traits	Slope	p-value of the slope	t-statistic of the slope	R^2
**Wild type**	Pole-to-pole final distance	0.75 ± 0.03	0.0000214	22.93	0.99
	Chromosome-to-chromosome final distance	0.09 ± 0.02	0.0184743	3.84	0.79
C27D9.1(RNAi)	Pole-to-pole final distance	0.81± 0.01	0.0000005	58.94	1.00
	Chromosome-to-chromosome final distance	0.12 ± 0.02	0.0014016	7.88	0.94
ani-2(RNAi)	Pole-to-pole final distance	0.73 ± 0.07	0.0003732	11.11	0.97
	Chromosome-to-chromosome final distance	0.08 ± 0.04	0.1135826	2.02	0.50

Table S5. Anaphase spindle microtubule quantification for different cell stages.

MT classes	Measurements	8-cell	16-cell	32-cell
**All MTs**		4094	1696	1937
**Interpolar MT**	Number of MTs	257	104	124
	Avg. MT length (µm) ± std	3.95 ± 0.75	3.55 ± 0.98	2.97 ± 0.77
**Mid-spindle MT**	Number of MTs	24	24	30
	Avg. MT length (µm) ± std	1.1 ± 0.6	1.92 ± 0.78	1.28 ± 0.56
**KMT**	Number of MTs	460	213	295
	Avg. MT length (µm) ± std	2.07 ± 0.5	1.32 ± 0.38	1.32 ± 0.39

Table S6| Analysis of variance (ANOVA) with Residual (Restricted) maximum likelihood using mixed linear model regression on wild-type spindle length.

**Mixed Linear Model Regression Results**						
**Model:**	MixedLM Dependent Variable:					Length
**No. observations:**	745		Method:			REML
**No. of groups:**	1		Scale:			0.5976
**Min. group size:**	745		Log-Likelihood:			-966.9404
**Max. group size:**	745		Converged:			Yes
**Mean group size:**	745.0					
	**Coefficient**	**Std. Err**	**z**	**P > |z|**	**CI [0.025]**	**CI [0.975]**
**Intercept**	14.275	1.736	8.222	0.000	10.872	17.677
**Cell Var**	0.355	0.087				
**Stage Var**	18.012	14.791				

z: Test statistic (Coefficient/Str. Err), **P:** *P*-value, **CI:** Confidence interval, **Var:** Variance.

REML: Residual (Restricted) maximum likelihood.

Table S7| Quantification of the poles elongation and the chromosome separation rates during and after laser ablation for the spindles at different embryonic cell stages in wild type.

Pole-to-pole: $D_{\text{P-P}}$ 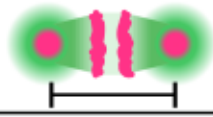				
	Stage	$\dot{D}_{\text{P-P(before)}}$ (μm/s)	$\dot{D}_{\text{P-P(after)}}$ (μm/s)	$\Delta\dot{D}_{\text{P-P}}$ (μm/s)
Control 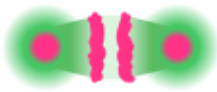	2-cell	0.077±0.049 (n=4)	0.062±0.039 (n=4)	-0.015±0.020 (n=4)
	4-cell	0.072±0.015 (n=7)	0.054±0.021 (n=8)	-0.018±0.018 (n=7)
	8cell	0.074±0.028 (n=8)	0.062±0.040 (n=7)	-0.005±0.054 (n=7)
	16-cell	0.058±0.025 (n=11)	0.042±0.027 (n=13)	-0.014±0.038 (n=11)
	16-cell+	0.065±0.036 (n=13)	0.037±0.021 (n=17)	-0.026±0.036 (n=13)
Cut btw P-C 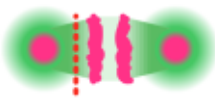	2-cell	0.103±0.050 (n=7)	0.691±0.179 (n=8)	0.605±0.181 (n=7)
	4-cell	0.096±0.007 (n=2)	0.246±0.142 (n=3)	0.231±0.004 (n=2)
	8cell	0.105±0.043 (n=4)	0.129±0.189 (n=5)	0.099±0.096 (n=4)
	16-cell	0.059±0.026 (n=13)	0.176±0.082 (n=13)	0.112±0.083 (n=12)
	16-cell+	0.090±0.029 (n=7)	0.134±0.059 (n=11)	0.041±0.076 (n=7)
Cut btw C-C 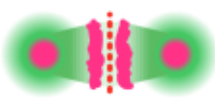	2-cell	0.084±0.024 (n=10)	0.638±0.161 (n=10)	0.555±0.144 (n=10)
	4-cell	0.080±0.020 (n=8)	0.386±0.224 (n=8)	0.305±0.234 (n=8)
	8cell	0.075±0.016 (n=5)	0.209±0.115 (n=5)	0.135±0.124 (n=5)
	16-cell	0.076±0.035 (n=18)	0.157±0.091 (n=16)	0.081±0.092 (n=16)
	16-cell+	0.069±0.039 (n=18)	0.098±0.071 (n=18)	0.029±0.080 (n=18)
Chromosome-to-chromosome: $D_{\text{C-C}}$ 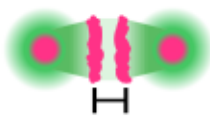				
	Stage	$\dot{D}_{\text{C-C(before)}}$ (μm/s)	$\dot{D}_{\text{C-C(after)}}$ (μm/s)	$\Delta\dot{D}_{\text{C-C}}$ (μm/s)
Control 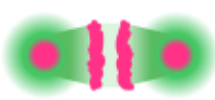	2-cell	0.122±0.005 (n=4)	0.074±0.007 (n=4)	-0.048±0.005 (n=4)
	4-cell	0.093±0.025 (n=9)	0.074±0.018 (n=9)	-0.018±0.014 (n=9)
	8cell	0.091±0.018 (n=8)	0.079±0.024 (n=8)	-0.012±0.021 (n=8)
	16-cell	0.089±0.034 (n=20)	0.065±0.025 (n=20)	-0.014±0.028 (n=18)
	16-cell+	0.100±0.028 (n=25)	0.068±0.022 (n=25)	-0.031±0.026 (n=25)
Cut btw P-C 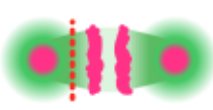	2-cell	0.136±0.008 (n=7)	0.064±0.033 (n=9)	-0.081±0.027 (n=7)
	4-cell	0.090±0.000 (n=1)	0.039±0.041 (n=3)	-0.031±0.000 (n=1)
	8cell	0.129±0.085 (n=4)	0.054±0.018 (n=6)	-0.082±0.076 (n=4)
	16-cell	0.094±0.035 (n=15)	0.050±0.022 (n=18)	-0.048±0.036 (n=15)
	16-cell+	0.118±0.035 (n=11)	0.035±0.021 (n=15)	-0.090±0.037 (n=10)
Cut btw C-C 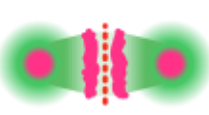	2-cell	0.106±0.022 (n=10)	0.745±0.187 (n=10)	0.639±0.182 (n=10)
	4-cell	0.106±0.017 (n=8)	0.412±0.210 (n=8)	0.306±0.204 (n=8)
	8cell	0.098±0.036 (n=6)	0.235±0.128 (n=6)	0.137±0.107 (n=6)
	16-cell	0.093±0.036 (n=25)	0.149±0.094 (n=25)	0.056±0.093 (n=25)
	16-cell+	0.116±0.030 (n=24)	0.145±0.083 (n=24)	0.029±0.081 (n=24)

Table S8| Light microscopy reporting table.

Hardware

**Instrument and Camera**	Custom-built Lattice Light Sheet microscope (LLSM) based on blue-prints provided by Betzig Lab[2,3], two Hamamatsu Orca Flash V4 cameras.
**Resolution**	Detection objective: Nikon 25x/1.1 water dipping objective.
**Filters and Light Sources**	MPB Communications 488 nm and 589 nm lasers. Dichroic mirror: Semrock BrightLine 560 nm longpass (FF560-FDi01), Emission filters: Semrock EdgeBasic 488 nm long-wave pass and 594 nm long-wave pass.

Quality Control

Is this the first report of the instrument? If yes, response to the below points is required, and providing validation data is encouraged.

Yes	☐
No	☒

For all instruments, confirm that the following quality control processes were run:

n/a	Confirmed	
☐	☐	Field homogeneity is assessed.
☐	☐	Illumination power stability is tested.
☐	☐	Stage drift is assessed.
☐	☐	Camera/detector noise is measured.

Methodology

**Sample preparation and imaging**	The imaged sample, *C. elegans* embryos, was extracted from adult hermaphrodite worms in M9 buffer and prepared for imaging, as described in the "Methods" section of this publication. The LLSM imaging of the embryos was done at a controlled temperature of 20°C.
**Fluorophores**	Centrosomes γ-tubulin is labeled with GFP; chromosome histones are labeled with mCherry; and the cell membrane PH domain is labeled with mKate2.<br><br>• GFP fluorophore was excited at 488 nm wavelength and detected at 530 nm wavelength.<br>• mCherry and mKate2 were excited at 589 nm and detected at 589 nm.<br><br>See Methods section "Imaging of C. elegans embryo" for more details.

Acquisition

**Software**	Custom LabVIEW Lattice Light Sheet software designed for Betzig Lab by Coleman Technologies, Inc., National Instruments[2]. Version: v 4.03724.0012 Built on: 1/18/2017 3:59:12 PM, rev 3724
**Enhancements**	None
**Imaging parameters**	25 ms exposure time, sequential imaging switching every z-stack.
**Size**	Size: 512 x 512 x 81 voxels. Voxel size: 0.1 µm x 0.1 µm x 0.5 µm. 81 planes at a step size of 0.5 um, moving both the light sheet and the detection objective.

Image Processing

**Software**	Custom Richardson-Lucy deconvolution algorithm in MATLAB developed by Betzig lab[2].
**Workflow**	A detailed description of the workflow, including the bioimage data post-processing after data acquisition, is provided in the Methods section of this publication.

References

1. Sulston, J. E., Schierenberg, E., White, J. G. & Thomson, J. N. The embryonic cell lineage of the nematode Caenorhabditis elegans. *Developmental Biology* **100**, 64–119 (1983).

2. Chen, B.-C. *et al.* Lattice light-sheet microscopy: Imaging molecules to embryos at high spatiotemporal resolution. *Science* **346**, 1257998 (2014).

3. Guillén-Boixet, J. *et al.* RNA-Induced Conformational Switching and Clustering of G3BP Drive Stress Granule Assembly by Condensation. *Cell* **181**, 346-361.e17 (2020).
