# Supplementary material for: Cell size reduction distinctly scales spindle elongation and chromosome segregation in C. elegans
Source: Nat Commun. 2026 Aug 6;17:7929. doi: 10.1038/s41467-026-76360-3 (PMC13448692; doi:10.1038/s41467-026-76360-3)
Supplement: Supplementary file 4 [file 41467_2026_76360_MOESM2_ESM.pdf]

Title: Supplementary Movie 1

Description: LLSM movie of a C. elegans wild-type embryo from the 2-cell to the 64-cell stage. The upper panel shows the two-dimensional view of the embryo to visualize the weak chromosome signal and the lower panel shows the maximum Z-projection view. The chromosomes (mCherry::histone, magenta), the cell membrane (mKate2::PH domain, magenta), and the centrosomes (GPF::γ-tubulin, green) are shown. Time resolution: 10.3 s.

Title: Supplementary Movie 2

Description: Movie of a 3D segmented and rendered mitotic C. elegans embryo in 32-cell stage. The one-frame movie was created using the data from the wide-type embryonic data. It shows the cells at different stages (interphase, prophase, metaphase, and anaphase) during development. The spindles in their corresponding cells are highlighted in green for the centrosomes and magenta for the chromosomes.

Title: Supplementary Movie 3

Description: LLSM movie of C. elegans ani-2(RNAi) embryo from the 2-cell to late embryonic cell stage. The upper panel shows the two-dimensional view of the embryo to visualize the weak chromosome signal, and the lower panel shows the maximum Z-projection view. The chromosomes (mCherry::histone, magenta), the cell membrane (mKate2::PH domain, magenta), and centrosomes (GPF::γ-tubulin, green) are shown. Time resolution: 10.3 s.

Title: Supplementary Movie 4

Description: LLSM movie of C. elegans C27D9.1(RNAi) embryo from the 2-cell to late 21 embryonic cell stage. The upper panel shows the two-dimensional view of the embryo to visualize the weak chromosome signal, and the lower panel shows the maximum Z-projection view. The Chromosomes (mCherry::histone, magenta), the cell membrane (mKate2::PH domain, magenta), and the centrosomes (GPF::γ-tubulin, green) are shown. Time resolution: 10.3 s.

Title: Supplementary Movie 5

Description: FESLA experiment with a wide-type C. elegans embryo. The laser cut (t = 0 sec) between the pole and chromosomes is indicated by two red arrows. Scale bar, 10 µm.

Title: Supplementary Movie 6

Description: Three-dimensional reconstruction by electron tomography showing an 8-cell stage anaphase spindle in a C. elegans wild-type embryo (corresponding to Fig. 3i (i-iii)). Stacked tomographic sections used for the reconstruction of the spindle and the chromosomes are shown. Astral microtubules (AMTs) are green, kinetochore microtubules (KMTs) orange, interpolar microtubules yellow/pink, mid-spindle microtubules red, the centrioles purple, and the chromosomes white.

Title: Supplementary Movie 7

Description: (Left panel) Three-dimensional visualization of the interpolar and the mid-spindle microtubules in the spindle midzone of an anaphase spindle at the 8-cell stage in a C. elegans wild-type embryo. Interpolar microtubules are shown in yellow/pink, mid-spindle microtubules are in red, centrioles are in purple, and chromosomes are in white. (Right panel) Three-dimensional visualization of interaction regions between interpolar and mid-spindle microtubules corresponding to the spindle shown in the left panel. Interpolar microtubules are shown in olive green, mid-spindle microtubules are in red, centrioles are in purple, and chromosomes are in white. Both movies correspond to Fig. 3i (iv).

Title: Supplementary Movie 8

Description: Three-dimensional reconstruction of a 16-cell stage anaphase spindle in a C. elegans embryo (corresponding to Fig. 3j (i-iii)). Stacked tomographic sections used for the reconstruction of the spindle and chromosomes are shown. AMTs are green, KMTs orange, interpolar microtubules yellow/pink, mid-spindle microtubules red, the centrioles purple, and chromosomes white.

Title: Supplementary Movie 9

Description: Three-dimensional reconstruction of a 16-cell stage anaphase spindle in a C. elegans 53 embryo. Left panel, Three-dimensional visualization of the interpolar and the mid-spindle microtubules in the spindle midzone of an anaphase spindle at the 16-cell stage in a C. elegans embryo. Interpolar microtubules are shown in yellow/pink, mid-spindle microtubules in red, centrioles in purple, and chromosomes in white. Right panel, Three-dimensional visualization of interaction regions between interpolar and mid-spindle microtubules corresponding to the spindle shown in the left panel. Interpolar microtubules are shown in olive green, mid-spindle microtubules are in red, centrioles are in purple, and chromosomes are in white. Both movies correspond to Fig. 3j (iv).

Title: Supplementary Movie 10

Description: Three-dimensional reconstruction of a 32-cell stage anaphase spindle in a C. elegans wild-type embryo (corresponding to Fig. 3k (i-iii)). Stacked tomographic

sections used for the reconstruction of the spindle and the chromosomes are shown. AMTs are green, KMTs orange, interpolar microtubules yellow/pink, mid-spindle microtubules red, the centrioles purple, and chromosomes white.

Title: Supplementary Movie 11

Description: (Left panel) Three-dimensional visualization of the interpolar and the mid-spindle microtubules in the spindle midzone of an anaphase spindle at the 32-cell stage in a C. elegans wild-type embryo. Interpolar microtubules are shown in yellow/pink, mid-spindle microtubules are in red, centrioles are in purple, and chromosomes are in white. (Right panel) Three-dimensional visualization of interaction regions between interpolar and mid-spindle microtubules corresponding to the spindle shown in the left panel. Interpolar microtubules are shown in olive green, mid-spindle microtubules are in red, centrioles are in purple, and chromosomes are in white. Both movies correspond to Fig. 3k (iv).

Title: Supplementary Data 1

Description: (mitotic spindle atlas) Extended supplementary data for the mitotic spindle atlas for Fig. 3. This folder contains subdirectories with datasets from quantified spindle length and chromosome dynamics across all cell types from the 2- to 64-cell stages, for each analyzed embryonic condition (wild type, ani-2(RNAi), and C27D9.1(RNAi) embryos). Each subfolder contains a spreadsheet of the results for the corresponding datasets.
